# Integrated Physiological and Transcriptomic Analyses Revealed Improved Cold Tolerance in Cucumber (*Cucumis sativus* L.) by Exogenous Chitosan Oligosaccharide

**DOI:** 10.3390/ijms24076202

**Published:** 2023-03-25

**Authors:** Chong Tan, Na Li, Yidan Wang, Xuejing Yu, Lu Yang, Ruifang Cao, Xueling Ye

**Affiliations:** College of Horticulture, Shenyang Agricultural University, 120 Dongling Road Shenhe District, Shenyang 110866, China

**Keywords:** cucumber, cold stress, exogenous substance, chitosan oligosaccharide, transcriptome

## Abstract

Cucumber (*Cucumis sativus* L.), sensitive to cold stress, is one of the most economically important vegetables. Here, we systematically investigated the roles of exogenous glycine betaine, chitosan, and chitosan oligosaccharide in alleviating cold stress in cucumber seedlings. The results showed that 50 mg·L^−1^ chitosan oligosaccharide had the best activity. It effectively increases plant growth, chlorophyll content, photosynthetic capacity, osmotic regulatory substance content, and antioxidant enzyme activities while reducing relative electrical conductivity and malondialdehyde levels in cucumber seedlings under cold stress. To reveal the protective effects of chitosan oligosaccharide in cold stress, cucumber seedlings pretreated with 50 mg·L^−1^ chitosan oligosaccharide were sampled after 0, 3, 12, and 24 h of cold stress for transcriptome analysis, with distilled water as a control. The numbers of differentially expressed genes in the four comparison groups were 656, 1274, 1122, and 957, respectively. GO functional annotation suggested that these genes were mainly involved in “voltage-gated calcium channel activity”, “carbohydrate metabolic process”, “jasmonic acid biosynthetic”, and “auxin response” biological processes. KEGG enrichment analysis indicated that these genes performed important functions in “phenylpropanoid biosynthesis”, “MAPK signaling pathway—plant”, “phenylalanine metabolism”, and “plant hormone signal transduction.” These findings provide a theoretical basis for the use of COS to alleviate the damage caused by cold stress in plant growth and development.

## 1. Introduction

The living environment has a restrictive effect on plant growth, considering its inherent properties. Environmental factors that are not conducive to plant growth are collectively referred to as adversities, and they are divided into biotic and abiotic stresses [1]. Cold stress is an abiotic factor that significantly affects plant growth, development, quality, yield, and geographical distribution [2,3,4].

Cold stress is divided into two categories: chilling (0–15 °C) and freezing (<0 °C) stress, which provoke different response mechanisms [5]. Chilling stress affects cell membrane fluidity, metabolism-related enzyme activities, reactive oxygen species (ROS) accumulation, and other physiological and biochemical activities. This reduces the plant metabolic rate, which in turn hinders plant growth and development. Freezing stress is more severe than cold stress, causing ice crystals to form within and between plant cells, which results in mechanical damage, cell rupture, and direct plant death [6]. In general, plants in the tropics and subtropics are less resistant to low temperatures than those in the temperate and cold zones. Upon exposure to a period of low but non-freezing temperatures, most temperate plants can enhance their freeze resistance through a process known as cold acclimation [7,8]. 

Plants have evolved complex regulatory networks to sense and respond to cold stress. However, how plants sense temperature decreases remain very inconclusive. At first, it is widely believed that the cell membrane is the primary site of temperature perception [9]. Accumulating evidence suggests that plants can sense temperature at the nucleus [10]. After sensing temperature stress signals, plants can transmit cold signals to cells via calcium (Ca^2+^) signaling or other secondary messengers [2]. Various Ca^2+^ channels, such as the transient receptor potential (TPR) family, function as temperature sensors in animals [11,12]. Although plants have no TRP-orthologous proteins, other calcium-permeable channels, such as cyclic nucleotide-gated ion channels (CNGCs), calcium-permeable mechanosensitive channels, such as Mid1-complementing activity 1 (MCA1) and 2 (MCA2), and calcium-permeable transporters, such as ANNEXIN 1 (ANN1) and 4 (ANN4), have been reported to mediate cold-induced cytosolic Ca^2+^ influx [13,14,15]. 

Multiple low-temperature responsive genes are activated and regulated at the transcriptional and post-transcriptional levels. The dehydration-response element-binding protein 1/C-repeat binding factors (DREB1/CBFs)-dependent signaling pathway is the subject of the most thorough research in the response to cold stress [16]. DREB1/CBFs are APETALA2/ethylene-responsive factor (AP2/ERF) transcription factors that directly activate the expression of cold-inducible genes, such as cold-regulated/responsive to dehydration (COR/RD) genes. Many upstream transcription factors have been reported to regulate the expression of DREB1/CBF genes. Calmodulin-binding transcription activator (CAMTA) and REVEILLEs (RVEs) are two of the major transcriptional activators. Circadian Clock Associated 1 (CCA1), Late Elongated Hypocotyl (LHY), and phytochrome-interacting factors (PIFs) act as transcriptional repressors of DREB1/CBF expression [17,18,19,20,21,22,23]. In addition to transcriptional regulation, the DREB1/CBF pathway is reported to be regulated at the post-transcriptional level [24,25,26,27,28,29,30]. Certainly, other signaling pathways can directly regulate the expression of COR/RD genes to achieve cold-stress tolerance without the need for the mediation of DREB1/CBF genes [31,32,33,34]. 

Cucumber (*Cucumis sativus* L.) is one of the most economically important vegetables cultivated worldwide. Cucumbers are typical chilling-sensitive species that are extremely vulnerable to cold stress during solar greenhouse cultivation in winter or early spring in northern China. Under cold stress, cucumbers grow slowly, wither, and even die, thus seriously affecting crop productivity and quality. Therefore, improving cold tolerance is of great theoretical and practical significance for cucumber breeding [35]. Exogenous substances, such as melatonin (MT), abscisic acid (ABA), jasmonic acid (JA), and salicylic acid (SA), are generally characterized by small amounts, quick effects, high benefits, and wide stress resistance. Additionally, they have been widely used to improve tolerance to cold stress [36,37,38]. In the present study, glycine betaine (GB), chitosan (CTS), and chitosan oligosaccharide (COS) were used as exogenous substances to investigate their positive effects in improving cold stress tolerance in cucumber seedlings. The most suitable exogenous substances and their optimal application concentrations were screened via analysis of physiological and biochemical indices. This provides the foundation for their reasonable popularization and application in cucumber production. Combined with transcriptome sequencing technology, this study provides a theoretical basis for the study of the molecular mechanisms of exogenous substance spray treatment to improve the low-temperature tolerance of cucumber. Notably, this is the first study to systematically analyze the important roles of exogenous GB, CTS, and COS in cold tolerance in cucumber.

## 2. Results

### 2.1. Effects of the Exogenous Substances on Cucumber Seedling Growth and Physiological Characteristics under Low-Temperature Stress

**Figure 1:**
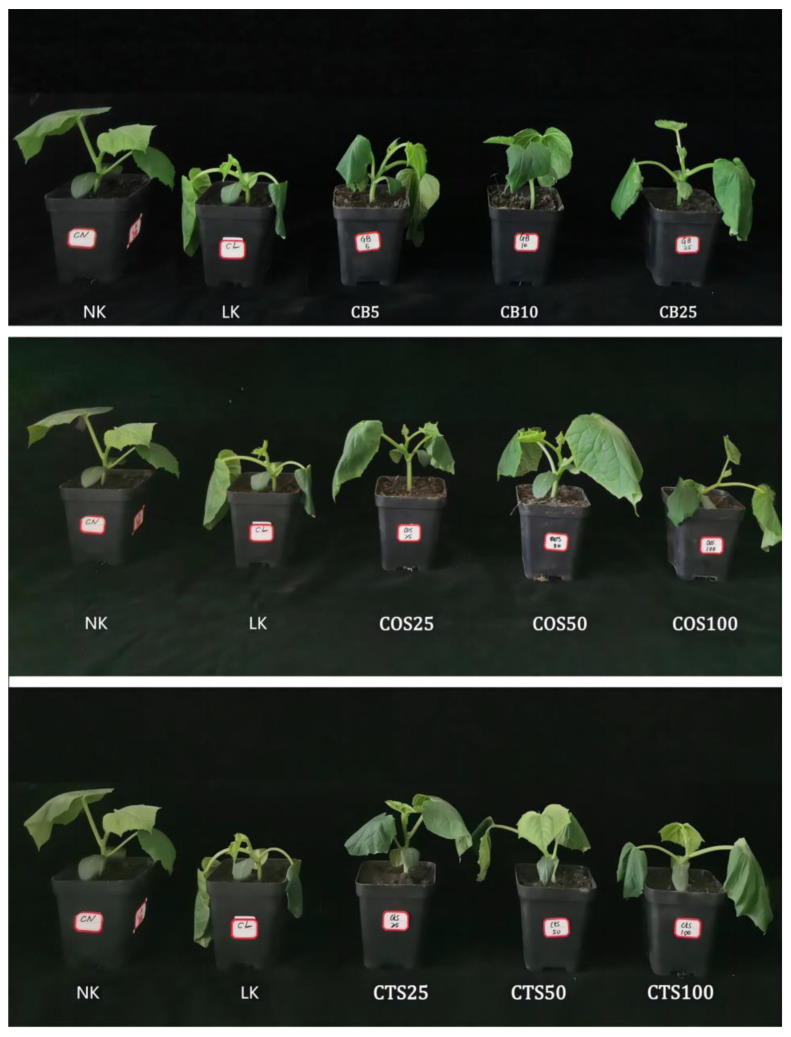
Phenotypic characteristics of cucumber seedlings treated with different exogenous substances under cold stress (12 °C/6 °C, day/night) after 24 h. NK group represents cucumber seedlings sprayed with distilled water and subjected to normal temperature (28 °C/18 °C, day/night). LK group represents cucumber seedlings sprayed with distilled water and subjected to cold stress. GB5, GB10, and GB25 groups represent cucumber seedlings sprayed with 5 mM, 10 mM, and 25 mM GB subjected to cold stress, respectively. The COS25, COS50, and COS100 groups represent cucumber seedlings sprayed with 25 mg·L^−1^, 50 mg·L^−1^, and 100 mg·L^−1^ COS subjected to cold stress, respectively. The CTS25, CTS50, and CTS100 groups represent cucumber seedlings sprayed with 25 mg·L^−1^, 50 mg·L^−1^, and 100 mg·L^−1^ CTS subjected to cold stress, respectively.

**Figure 2:**
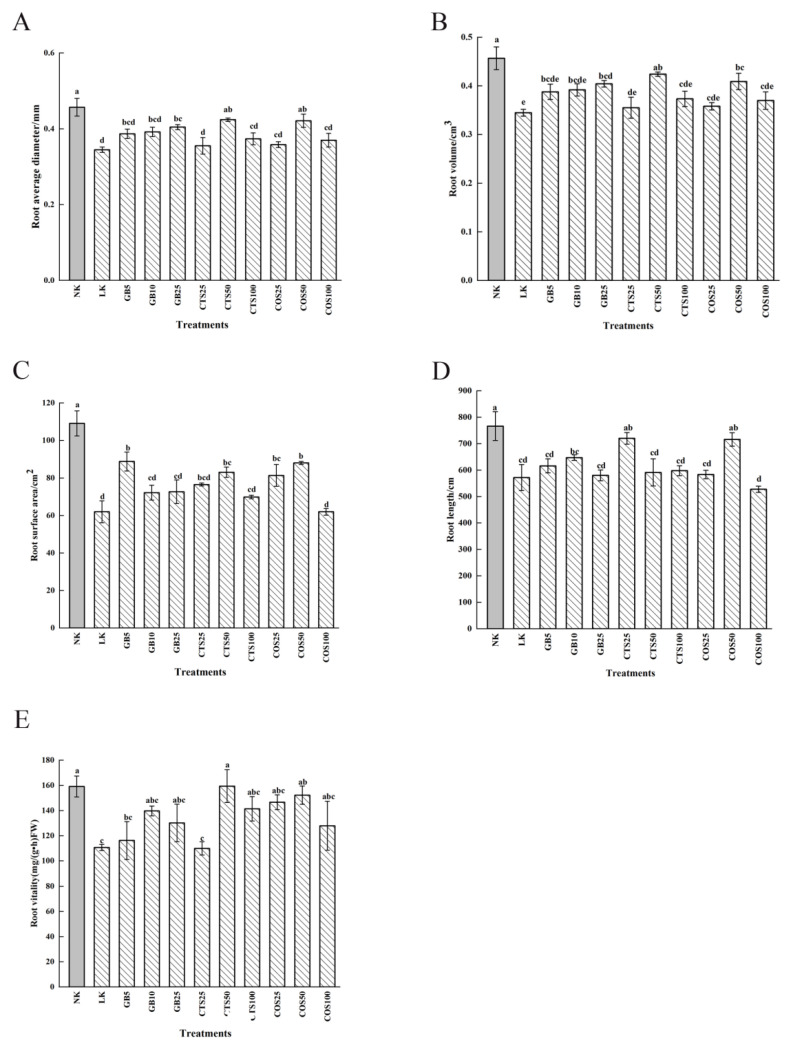
Effects of exogenous substances on (**A**) root average diameter, (**B**) root volume, (**C**) root surface area, (**D**) root length, and (**E**) root vitality of cucumber seedlings under cold stress. Different letters indicate significant differences at *p* < 0.05.

After 24 h of low-temperature stress, the leaves of cucumber seedlings treated in the LK group (distilled water-sprayed group, cold stress) were severely wilted, with extensive waterlogging on the abaxial leaf side and a higher angle of stem droop compared to those in the NK group (distilled water-sprayed group, normal temperature). Compared to the LK group, the seedlings treated with exogenous substances exhibited significantly reduced cold damage (Figure 1). The plant height, stem diameter, above- and below-ground dry weights, and fresh weights of cucumber seedlings in the LK group were significantly reduced after 120 h of low-temperature stress compared with those in the NK group (Table 1). With an increase in exogenous substance spray concentration, the relative increases in cucumber seedlings treated with the exogenous substance spray showed a trend of initially increasing and then decreasing, compared with those in the LK group. Compared with the LK group, the plant height, stem diameter, above-ground dry weight, above-ground fresh weight, below-ground dry weight, and below-ground fresh weight increased by 20.30%, 21.6%, 12.81%, 15.09%, 35.92%, and 42.85%, respectively, in the GB10 group (10 mM GB-sprayed group, cold stress), and increased by 5.04%, 21.91%, 2.26%, 3.77%, 26.21%, and 28.57%, respectively, in the CTS50 group (50 mg·L^−1^ CTS-sprayed group, cold stress). Furthermore, the plant height, stem diameter, above-ground dry weight, above-ground fresh weight, below-ground dry weight, and below-ground fresh weight also increased by 13.31%, 23.15%, 14.12%, 16.98%, 35.92%, and 28.57%, respectively, in the COS50 group (50 mg·L^−1^ COS-sprayed group, cold stress), compared to that of the LK group. After 120 h of low-temperature stress, the average root diameter, volume, surface area, length, and vitality of cucumber seedlings treated with and without exogenous substances decreased compared with those in the NK group. Compared with the LK group, the GB25 (10 mM GB-sprayed group, cold stress), CTS50, and COS50 groups showed significant differences in root average diameter, which increased by 19.2%, 23.01%, and 22.1%, respectively (Figure 2A). The GB25, CTS50, and CTS50 groups showed significant differences in root volume compared to those in the LK group, with increases of 17.3%, 23.01%, and 18.65%, respectively (Figure 2B). The GB5 (5 mM GB-sprayed group, cold stress), CTS50, and COS50 groups showed significant differences in root surface area compared with those in the LK group, with increases of 43.27%, 34.01%, and 42.02%, respectively (Figure 2C). The GB10, CTS25 (25 mg·L^−1^ CTS-sprayed group, cold stress), and COS50 groups showed significant differences in root length compared with those in the LK group, with increases of 13.23%, 25.97%, and 25.28%, respectively (Figure 2D). The CTS50 and COS50 groups showed significant differences in root vitality compared with those in the LK group, with increases of 44.14% and 37.60%, respectively (Figure 2E).

**Figure 3:**
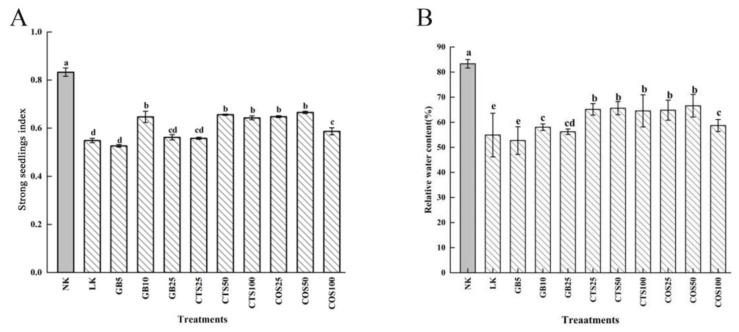
Effects of exogenous substances on (**A**) the strong seedling index and (**B**) the relative water content of cucumber seedlings under cold stress. Different letters indicate significant differences at *p* < 0.05.

When exposed to low-temperature stress for 120 h, the strong seedling index and relative water content of cucumber seedlings, following treatments in the LK group and the group treated with exogenous substances, decreased compared with those in the NK group (Figure 3). The GB10, CTS50, and CTS100 (100 mg·L^−1^ CTS-sprayed group, cold stress), COS25 (25 mg·L^−1^ COS-sprayed group, cold stress), COS50, and COS100 (100 mg·L^−1^ COS-sprayed group, cold stress) groups all showed significant differences in the strong seedling index compared with those in the LK group. Among them, the GB10, CTS50, and COS50 groups showed increases of 17.85%, 19.49%, and 21.26%, respectively (Figure 3A). Compared to the LK group, the CTS25, CTS50, CTS100, COS25, and COS50 groups showed significant differences in relative water content, among which the CTS50 and COS50 groups showed increases of 19.49% and 21.25%, respectively (Figure 3B). 

**Figure 4:**
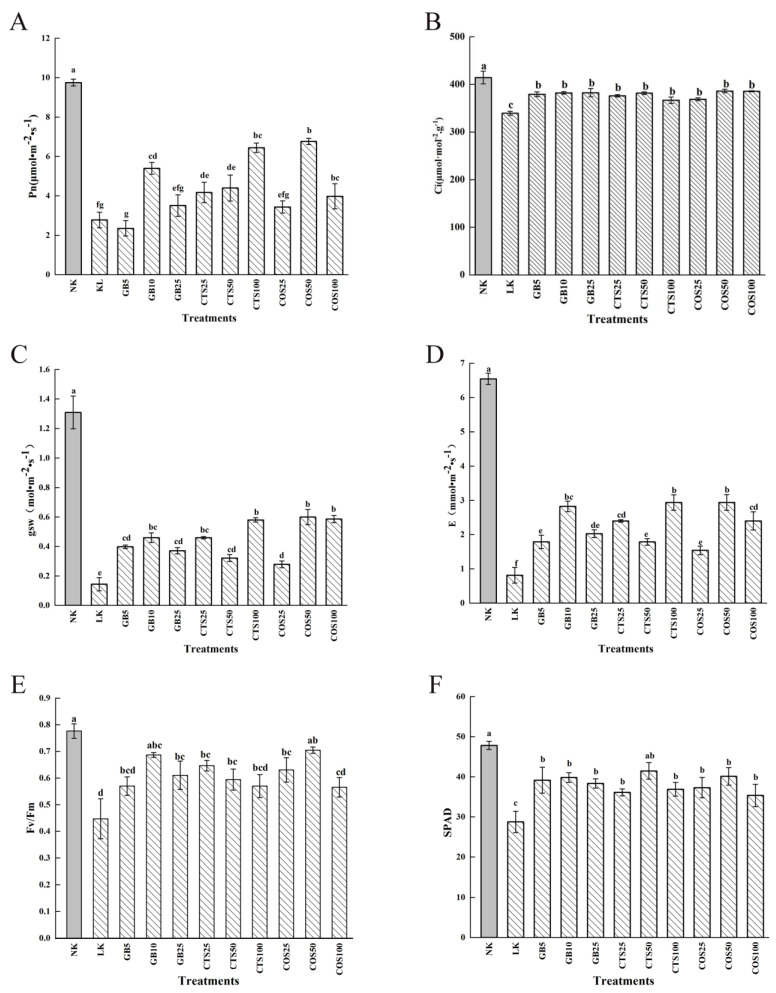
Effects of exogenous substances on (**A**) net photosynthesis (Pn), (**B**) intercellular CO_2_ concentration (Ci), (**C**) stomatal conductance (gsw), (**D**) transpiration rate (E), (**E**) chlorophyll fluorescence parameters (Fv/Fm), and (**F**) SPAD of cucumber seedlings under cold stress. Different letters indicate significant differences at *p* < 0.05.

After 24 h of low-temperature stress, intercellular CO_2_ concentration (Ci), stomatal conductance (gsw), net photosynthesis (Pn), transpiration rate (E), and chlorophyll fluorescence parameters (Fv/Fm) were all reduced in the LK and exogenous substance application groups compared to those in the NK group (Figure 4). Under low-temperature stress, the GB10, CTS25, CTS50, CTS100, COS50, and COS100 groups showed significant differences in Pn compared with those in the LK group, among which GB10, CTS100, and COS50 increased by 34.87%, 35.83%, and 36.55%, respectively (Figure 4A). The Ci of cucumber seedlings treated with exogenous spray under low-temperature stress was higher than that of the LK group. Furthermore, the GB10, GB25, CTS25, CTS50, COS25, and COS50 groups showed significant differences in Ci compared to the LK group, among which the GB10, CTS50, and COS50 groups showed increases of 12.60%, 12.42%, and 13.75%, respectively (Figure 4B). The gsw of cucumber seedlings treated with exogenous substances was greater than that of LK seedlings. All seedlings that underwent exogenous substance treatment showed significant differences in gsw compared with those in the LK group, among which the GB10, CTS100, and COS50 groups showed increases of 36.0%, 52.0%, and 49.8%, respectively (Figure 4C). Similarly, all seedlings treated with an exogenous substance showed significant differences in E compared with those in the LK group, among which the GB10, CTS100, and COS50 showed increases of 57.1%, 60.11%, and 58.7%, respectively (Figure 4D). The GB10, GB25, CTS25, CTS50, COS25, and COS50 groups showed significant differences in Fv/Fm compared with the LK group, among which the GB10, CTS25, and COS50 groups showed increases of 34.88%, 44.58%, and 57.62%, respectively (Figure 4E).

After 120 h of low-temperature stress, the SPAD values in the leaves of the LK group decreased significantly compared with those of the NK group. The SPAD value after treatment with exogenous substances was higher than that of the LK group, and the SPAD value first increased and then decreased with an increase in the concentration of exogenous substances. All seedlings treated with the exogenous substance showed significant differences in the SPAD value compared with that of the LK group, among which the GB10, CTS50, and COS50 groups showed increases of 38.52%, 43.17%, and 39.43%, respectively (Figure 4F).

In conclusion, the analysis of cucumber seedlings' growth and physiological indices showed that the GB10, COS50, and CTS50 groups exhibited the most noticeable effect on alleviating low-temperature injury under cold stress.

### 2.2. Effects of the Exogenous Substances at Optimum Concentration on Cold Tolerance of Cucumber Seedlings

**Figure 5:**
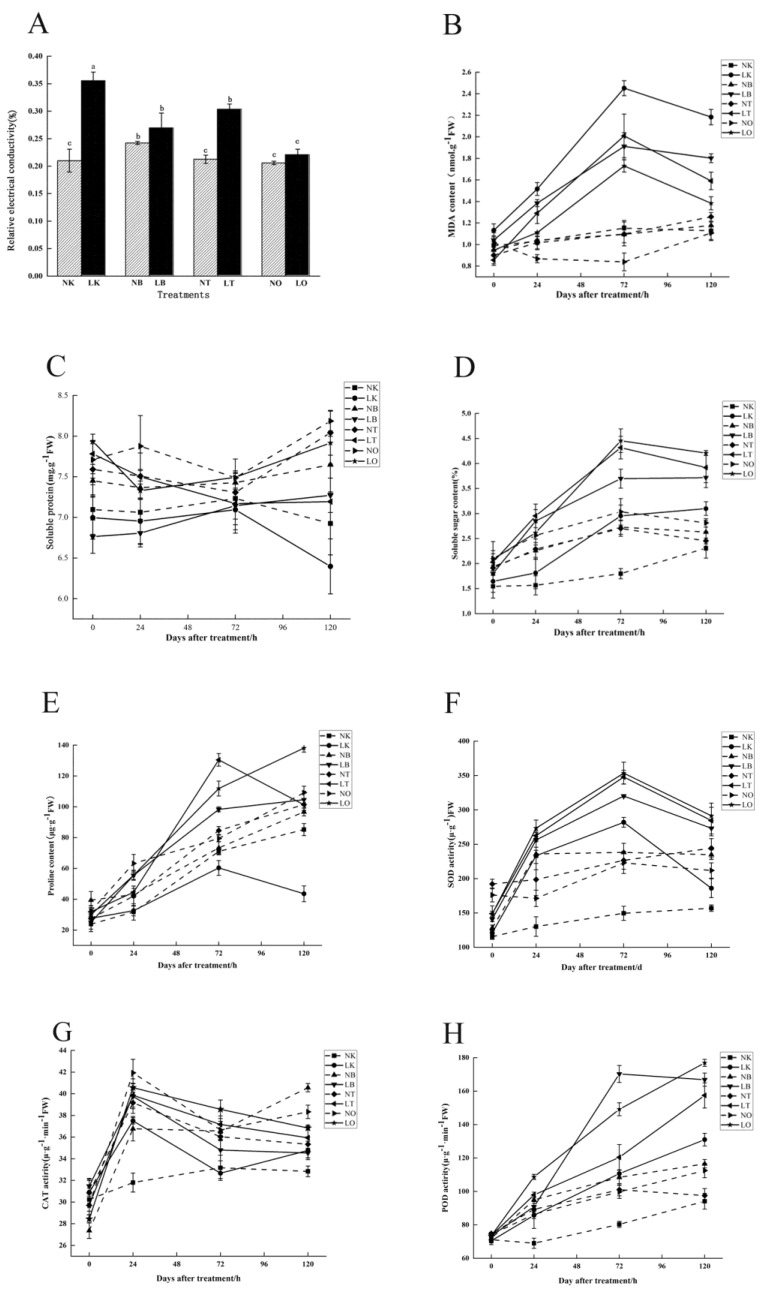
Effects of exogenous substances on (**A**) relative electrical conductivity (REC), (**B**) malondialdehyde (MDA), (**C**) soluble protein (SP), (**D**) soluble sugar (SS), (**E**) proline (Pro), (**F**) superoxide dismutase (SOD), (**G**) catalase (CAT), and (**H**) peroxidase (POD) of cucumber seedlings under cold stress. Different letters indicate significant differences at *p* < 0.05.

After the preliminary concentration screening, it was found that GB10, CTS50, and COS50 treatments significantly improved the low-temperature tolerance of cucumber seedlings. The relative electrical conductivity (REC) value showed no significant difference between the NK and NT (50 mg·L^−1^ CTS-sprayed group, normal temperature) and NO (50 mg·L^−1^ COS-sprayed group, normal temperature) treatments. However, the results of the NB group (10 mM GB-sprayed group, normal temperature) were significantly different from those of the other three treatment groups. The REC values of the LB (10 mM GB-sprayed group, cold stress), LT (50 mg·L^−1^ CTS-sprayed group, cold stress), and LO (50 mg·L^−1^ COS-sprayed group, cold stress) groups showed significant differences compared to those of the LK group. The REC values of the LB, LT, and LO treatment groups were decreased by 24.24%, 14.61%, and 38.00%, respectively, compared with those of the LK group (Figure 5A). The malondialdehyde (MDA) content in cucumber seedlings first increased and then decreased with the extension of low-temperature stress. The MDA content was the highest at 72 h under low-temperature stress; it then decreased at 120 h but was still higher than its initial value at 24 h. Compared with the LK group, MDA content after 72 h of low-temperature stress in the LB, LT, and LO groups decreased by 18.49%, 14.3%, and 26.8%, respectively (Figure 5B). With the extension of treatment time, the soluble protein (SP) content of each treatment group showed no significant change. However, after 120 h of low-temperature treatment, there was a significant difference between the different treatment groups. After 120 h of low-temperature stress, the SP content in the LT, LB, and LO groups increased by 13%, 15%, and 33%, respectively, compared to that of the LK group (Figure 5C). The soluble sugar (SS) content of cucumber seedlings in each treatment group increased and then decreased with the extension of time after low-temperature stress. The SS content reached its highest concentration at 72 h and decreased at 120 h, but it was higher than that at 24 h. After 72 h of low-temperature stress, the SS content in the LB, LT, and LO groups increased by 82%, 110%, and 112%, respectively, compared to those in the LK group (Figure 5D). Under low-temperature stress, proline (Pro) content in each treatment group showed an increasing trend with increasing treatment time. After 120 h of low-temperature stress, Pro content in the LO and LB groups continued to increase, with the LT group exhibiting the highest Pro content concentration at 72 h. After 120 h of low-temperature stress, Pro content in LB, LT, and LO increased by 127.9%, 120.7%, and 163.6%, respectively, compared with that in the LK group (Figure 5E).

Over time, superoxide dismutase (SOD) activity in the LT, LO, and LB groups was higher than that in the LK group. After 72 h of low-temperature stress, SOD activity reached its maximum among all treatments and decreased after 120 h. Compared with the LK group, the LB, LT, and LO groups exhibited increased SOD activity after 72 h of low-temperature stress, by 66.92%, 83.3%, and 89.5%, respectively (Figure 5F). Catalase (CAT) activity reached its highest value after 24 h of treatment and then began to decline. After 24 h of low-temperature stress, there was no significant difference between the treatment groups, and the differences only reached a significant level at 72 h. Compared with that of the LK group, CAT activity after 72 h of low-temperature stress in the LB, LT, and LO groups increased by 12.9%, 17.2%, and 19.7%, respectively (Figure 5G). With the extension of the low-temperature treatment time, peroxidase (POD) activity gradually increased. Under low-temperature stress, the degree of damage to the membrane system was reduced by increasing POD activity. The change range of the LB group was the largest, reaching its highest at 72 h before decreasing while being on the rise in the LT and LO groups. Compared to that of the LK group, POD activity after 120 h of low-temperature stress in the LB, LT, and LO groups increased by 65.3%, 41.5%, and 76.9%, respectively (Figure 5H).

In conclusion, the above results indicate that LO treatment, that is, the administration of an exogenous spray of 50 mg·L^−1^ COS at low temperature, has the best effect on alleviating low-temperature stress.

### 2.3. Transcriptome Analysis of Exogenous COS in Response to Cold Stress in Cucumber Seedlings

Consequently, 50 mg·L^−1^ COS was selected as the exogenous substance to spray cucumber seedlings for transcriptome sequencing analysis. After being exposed to cold stress for 0, 3, 12, and 24 h, cucumber seedlings sprayed with COS were collected as treatment groups and named LC0 (50 mg·L^−1^ COS-sprayed group, cold stress 0 h), LC3 (50 mg·L^−1^ COS-sprayed group, cold stress 3 h), LC12 (50 mg·L^−1^ COS-sprayed group, cold stress 12 h), and LC24 (50 mg·L^−1^ COS-sprayed group, cold stress 24 h), respectively. Cucumber seedlings sprayed with distilled water under the same conditions were used as controls and named L0 (distilled water-sprayed group, cold stress 0 h), L3 (distilled water-sprayed group, cold stress 3 h), L12 (distilled water-sprayed group, cold stress 12 h), and L24 (distilled water-sprayed group, cold stress 24 h), respectively. Twenty-four libraries were constructed from eight samples (three biological replicates for each sample) (Table 2). Each library contained approximately 43.36 million raw reads (a total of 1040.72 million raw reads). After removing low-quality reads, 1015.96 million valid reads were obtained, with an average of 42.33 million valid reads per library, which represented an average of 97.62% of the raw reads. In all libraries, the Q30 values were over 97% and the GC content was over 42%. Approximately 97.61% of valid reads were mapped to the reference genome. Approximately 35.43 million reads per library could only be uniquely aligned to one location in the genome. Approximately 16,620 genes were identified in each library (Table S1). This suggests that the RNA-Sequencing (RNA-Seq) data were of robust quality and reliable results were obtained from the transcriptome assembly.

**Figure 6:**
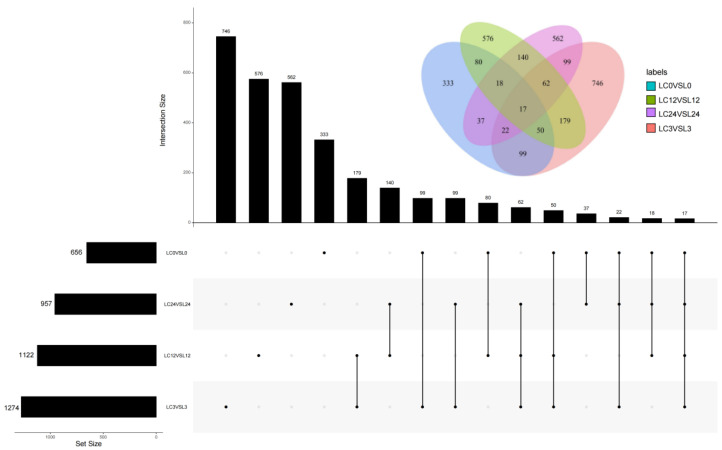
UpSet diagram of DEGs in the four pairwise comparisons (LC0 vs.L0, LC3 vs. L3, LC12 vs. L12, and LC24 vs. L24). The left bar represents the raw number of each group, and the upper bar represents the number of intersections among the groups.

To comprehensively investigate differentially expressed genes (DEGs) in cucumber seedlings sprayed with 50 mg·L^−1^ COS in response to cold stress, fragments per kilobase of exon per million mapped fragments (FPKM) was used to calculate gene expression. It was based on the criteria of *p* < 0.05 for the adjusted *p*-value and greater than 1 for the log2 base of fold-change. After 0, 3, 12, and 24 h of cold stress, 656 (417 upregulated and 239 downregulated), 1274 (621 upregulated and 653 downregulated), 1122 (958 upregulated and 164 downregulated), and 957 (698 upregulated and 259 downregulated) DEGs were identified in the LC0 vs. L0, LC3 vs. L3, LC12 vs. L12, and LC24 vs. L24 pairwise comparisons, respectively (Tables S2–S5, respectively; Figure 6). Figure 6 shows that 17 common genes were detected among the four comparison groups. This suggests that these genes are involved in the response to cold stress after exogenous COS spraying. In addition, a large number of DEGs were generated in the other three comparisons compared with the LC0 vs. L0 pairwise comparison. A higher number of unique DEGs was generated in the LC3 vs. L3 pairwise comparisons. To verify the accuracy of the RNA-Seq data, we randomly selected 20 DEGs in the phenylalanine metabolic pathway, phenylpropanoid biosynthesis, ABC transporter, MAPK signaling pathway—plant, plant hormone signal transduction, and plant–pathogen interactions for qRT-PCR validation. The results between the qRT-PCR and RNA-Seq data were highly consistent. This suggests that the RNA-Seq data are highly credible for use in the functional enrichment analysis (Figure S1).

To explore the potential functions of DEGs produced by spraying COS under low-temperature stress, we performed GO analysis. In the LC0 vs. L0 pairwise comparison, a total of 375 DEGs were classified into 296 GO terms (Table S6). The GO significant enrichment analysis identified 52 items at an adjusted *p* < 0.05, and the top 20 GO significant enrichment terms are displayed in the scatter diagram (Table S6 and Figure S2A). In particular, “voltage-gated calcium channel activity” and “carbohydrate metabolic process” were significantly enriched in biological processes. In the LC3 vs. L3 pairwise comparison, 775 DEGs were classified into 296 GO terms (Table S7). The GO significant enrichment analysis identified 55 items at an adjusted *p* < 0.05, and the top 20 GO significant enrichment terms are displayed in the scatter diagram (Table S7 and Figure S2B). In particular, the “carbohydrate metabolic process” and “jasmonic acid biosynthetic process” were significantly enriched in biological processes. In the LC12 vs. L12 pairwise comparison, 724 DEGs were classified into 387 GO terms (Table S8). The GO significant enrichment analysis identified 41 items at an adjusted *p* < 0.05, and the top 20 GO significant enrichment terms are displayed in the scatter diagram (Table S8 and Figure S2C). “Response to auxin” in biological processes, in particular, was significantly enriched. In the LC24 vs. L24 pairwise comparison, 591 DEGs were classified into 366 GO terms (Table S9). The GO significant enrichment analysis identified 41 items at an adjusted *p* < 0.05, and the top 20 GO significant enrichment terms are displayed in the scatter diagram (Table S9 and Figure S2D). In particular, the “regulation of auxin polar transport” in biological processes was significantly enriched.

To explore the metabolic pathways of DEGs produced by spraying COS under low-temperature stress, we performed a KEGG analysis. In the LC0 vs. L0 pairwise comparison, 215 DEGs were classified into 93 metabolic pathways (Table S10). The KEGG significant enrichment analysis identified 7 pathways with adjusted *p* < 0.05, and the top 20 KEGG significant enrichment terms are displayed in a scatter diagram (Table S10 and Figure S3A). Of note, “phenylpropanoid biosynthesis (ko00940)” was significantly enriched in the LC0 vs. L0 pairwise comparison. In the LC3 vs. L3 pairwise comparison, 425 DEGs were classified into 109 metabolic pathways (Table S11). The KEGG significant enrichment analysis identified 7 pathways with adjusted *p* < 0.05, and the top 20 KEGG significant enrichment terms are displayed in a scatter diagram (Table S11 and Figure S3B). More importantly, “phenylpropanoid biosynthesis (ko00940)”, “MAPK signaling pathway—plant (ko04016)”, and “phenylalanine metabolism (ko00360)” were significantly enriched in the LC3 vs. L3 pairwise comparison. In the LC12 vs. L12 pairwise comparison, 346 DEGs were classified into 101 metabolic pathways (Table S11). The KEGG significant enrichment analysis identified 9 pathways with adjusted *p* < 0.05, and the top 20 KEGG significant enrichment terms are displayed in the scatter diagram (Table S12 and Figure S3C). Notably, “phenylalanine metabolism (ko00360)”, “phenylpropanoid biosynthesis (ko00940)”, “plant hormone signal transduction (ko04075), ” and “MAPK signaling pathway—plant (ko04016)” were significantly enriched in the LC12 vs. L12 pairwise comparison. In the LC24 vs. L24 pairwise comparison, 313 DEGs were classified into 107 metabolic pathways (Table S13). The KEGG significant enrichment analysis identified 6 pathways with adjusted *p* < 0.05, and the top 20 KEGG significant enrichment terms are displayed in a scatter diagram (Table S13 and Figure S3D). More importantly, “plant hormone signal transduction (ko04075)” and “phenylpropanoid biosynthesis (ko00940)” were significantly enriched in the LC24 vs. L24 pairwise comparison. 

**Figure 7:**
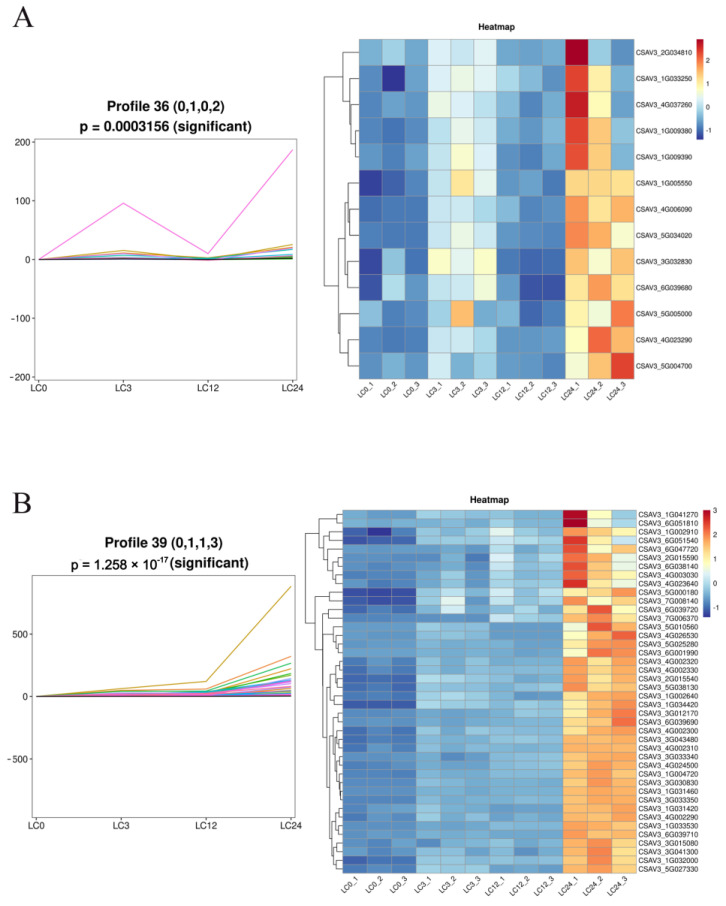
Patterns of gene expression across four treatments (LC0, LC3, LC12, and LC24). (**A**) The expression tendency and heatmap of the DEGs in profile 36. (**B**) The expression tendency and heatmap of the DEGs in profile 39.

We further analyzed the gene expression patterns of the DEGs significantly enriched in “phenylpropanoid biosynthesis”, “MAPK signaling pathway—plant”, “phenylalanine metabolism”, and “plant hormone signal transduction” in the four pairwise comparisons (LC0 vs. L0, LC3 vs. L3, LC12 vs. L12, and LC24 vs. L24). A total of 185 DEGs displayed considerable differences across LC0, LC3, LC12, and LC24, and these genes were clustered into 48 profiles (0–48) based on their expression patterns using the Short Time-series Expression Miner (STEM) software (Figure S4 and Table S14). The most represented clusters were profiles 36 and 39 (*p* < 0.05). In profile 36, the expression of 13 DEGs increased at LC3, decreased at LC12, and increased at LC24 (Figure 7A). In profile 39, the expression of 42 DEGs increased at LC3, remained relatively stable at LC12, and significantly increased at LC24 (Figure 7B). In brief, a large number of genes in different pathways were involved in the cold tolerance mechanism of cucumber seedlings after exogenous COS spraying.

## 3. Discussion

Cucumber originated in tropical areas, and cold stress severely restricts cucumber growth and yield. Chemical regulation of plant cold tolerance has been receiving increasing attention because it is generally considered to be a safe and affordable solution. Exogenous substances such as ABA, JA, MT, SA, and polyamines have certain application values in improving the cold tolerance of plants. In a previous study of cucumber, MT application enhanced cold tolerance by regulating the metabolism of PAs and ABA [39]. Exogenous selenite was shown to effectively alleviate cold stress and increase endogenous MT levels in cucumbers [40]. Endogenous 2,4-epibrassionolide (EBR) and SA application were reported to improve photosynthesis and cold tolerance in cucumber seedlings [41]. This study systematically analyzed the important role of exogenous GB, CTS, and COS in cold tolerance in cucumber for the first time. As an important osmoprotectant and antioxidant, GB improves plant resistance to abiotic stresses [42]. It has long been reported that exogenous GB can increase chilling- and freeze-tolerance in plants [43,44,45,46]. Exogenous GB can effectively alleviate the inhibitory effect of cold stress on tomato seed germination [47]. Reportedly, CTS is a deacetylated derivative of chitin and can also be used as an exogenous substance to improve plant tolerance to abiotic stress [48]. It has been reported that the exogenous application of CTS can improve plant tolerance to drought [49,50,51], salinity [52], and osmotic stresses [53]. Furthermore, COS, a degradation product of chitin/chitosan, was shown to promote plant growth and yield by enhancing plant resistance to drought, salinity, and toxic metal stresses [54,55,56,57]. As shown in Table 1 and Figure 2, Figure 3, Figure 4, Figure 5, physiological and biochemical indices indicated that the alleviation effect of GB, CTC, and COS on cold stress was concentration-dependent, and 10 mM GB, 50 mg·L^−1^ CTS, and 50 mg·L^−1^ COS were the optimal concentrations for each. Further analysis indicated that 50 mg·L^−1^ COS exhibited the best activity and could improve plant resistance to cold stress by promoting antioxidant enzyme activities, which was consistent with the findings of previous studies [56].

The mitogen-activated protein kinase (MAPK) cascade pathway is an important signal transduction pathway involved in abiotic stress responses. The MAPK cascade signaling pathway consists of three components: MAPK, MAPK kinase (MAPKK/MEK), and MAPK kinase kinase (MAPKKK/MEKK) [58]. CRLK1 can interact with MEKK1, and phosphorylation activates the MEKK1-mediated MAPK pathway MEKK1-MKK2-MPK4/6, thereby enhancing plant cold tolerance [59]. In *Arabidopsis*, MPK4 is a downstream component of hydrogen sulfide (H2S)-related cold stress resistance [60]. MPK3/6 can be activated by MKK4/5, which phosphorylates downstream ICE1 and destabilizes it, reducing plant tolerance to low temperatures [61,62]. The genome-wide analysis identified 14 MAPKs, 6 MAPKKs, and 59 MAPKKKs in cucumbers [63]. In our study, we found that MAPKKK (CsaV3_6G043320) was differentially expressed in LC0 vs. L0 and LC24 vs. L24 pairwise comparisons; MAPKKs (CsaV3_3G031180) were differentially expressed in LC3 vs. L3 pairwise comparisons; and MAPK (CsaV3_1G033530) was differentially expressed in LC3 vs. L3 and LC12 vs. L12 pairwise comparisons (Tables S2–S5). Among them, CsaV3_6G043320 was clustered in the most represented profile 39 (*p* < 0.05) across LC0, LC3, LC12, and LC24 (Figure 7B). This suggests that this gene may have a unique role in exogenous COS application to improve the cold tolerance of cucumber seedlings.

Phenolics are an essential class of plant secondary metabolites that are important in several metabolic and physiological processes in plants. Phenolics originating from phenylalanine are also known as phenylpropanoids [64]. In the phenylpropanoid pathway, phenylalanine is sequentially catalyzed by phenylalanine ammonia lyase (PAL), cinnamate 4-hydroxylase (C4H), and 4-Coumarate-CoA ligase (4CL), finally produces 4-Coumarate-CoA. Then, it enters the downstream synthesis pathway through different catalytic reactions to produce metabolites, such as lignin, anthocyanin, proanthocyanidin, and flavonoids [65]. In response to abiotic stress, plants activate the phenylpropanoid pathway to accumulate polyphenols, which help improve plant tolerance to various stress conditions such as heavy metals, salinity, drought, temperature, pesticides, and UV radiation. Under low-temperature stress, the increase in the amount of polyphenols, especially lignin, is due to the upregulated expression of PAL, cinnamyl alcohol dehydrogenase (CAD), and hydroxycinnamoyl transferase (HCT), which are crucial in protecting tobacco against cold stress [66]. Additionally, PAL is the first enzyme in the phenylpropanoid pathway, constituting the hub of primary metabolism and the phenylpropane metabolic pathway. In our study, the expression of five (CsaV3_4G002290, CsaV3_4G002300, CsaV3_4G002310, CsaV3_4G002320, and CsaV3_4G002330), two (CsaV3_6G039680 and CsaV3_6G039720), and two (CsaV3_6G039690 and CsaV3_6G039710) PAL genes was upregulated in the LC12 vs. L12, LC24 vs. L24, and in both the LC12 vs. L12 and LC24 vs. L24 pairwise comparisons, respectively (Tables S2–S5). Of the 9 PAL genes, all except CsaV3_6G039680 were clustered in the most represented profile 39 (*p* < 0.05) across LC0, LC3, LC12, and LC24 (Figure 7B). In conclusion, exogenous COS could improve cold tolerance by increasing the activities of key enzyme PALs of phenolic biosynthetic pathways in cucumber seedlings.

The TIFY family is defined by the core motif TIF[F/Y]XG, located in the TIFY domain, which can be divided into four subfamilies: ZIM-like (ZML), PEAPOD (PPD), jasmonate-ZIM-domain (JAZ), and TIFY [67]. TIFY proteins play important roles in the regulation of plant growth and development, as well as in the response to stress and phytohormone treatments. In terms of abiotic stresses, the TIFY family participates in the response to salt, drought, cold, and heavy metal stress [67,68,69]. TIFY genes have been identified in a variety of species, such as those of *Arabidopsis,* rice, poplar, tomato, and watermelon, but they have not been reported in cucumber [70]. In our study, the expression of two (CsaV3_6G051810 and CsaV3_1G041270) and one (CsaV3_3G030830) TIFY genes was upregulated in the LC24 vs. L24 and in both the LC12 vs. L12 and the LC24 vs. L24 pairwise comparisons, respectively (Tables S2–S5). All 3 TIFY genes clustered in the most represented profile 39 (*p* < 0.05) across LC0, LC3, LC12, and LC24 (Figure 7B). Previous reports have shown that TIFY family members, particularly JAZ subfamily proteins, play pivotal roles in the crosstalk between JA and other phytohormones [70]. Therefore, we conclude that exogenous COS could improve the response to cold stress by promoting the expression of TAPY genes, which have multiple regulatory roles in cell signal transduction and plant stress response regulation. 

The plant hormone ABA plays a key role in plant development and adaptation to stress [71]. Stress-induced ABA is sensed by the pyrabactin resistance 1-like (PYR/PYL) protein (hereafter referred to as PYLs), leading to the interaction of PYLs with clade A type 2C protein phosphatases (PP2Cs). The formation of PYL-ABA-PP2Cs ternary complexes inhibits PP2C dephosphorylation and releases sucrose non-fermenting 1-related protein kinase 2 proteins (SnRK2s). SnRK2s phosphorylate downstream transcription factors, such as ABA-responsive element-binding factors (ABFs), to regulate the expression of ABA-responsive genes [72]. Furthermore, PYLs are the core regulators of ABA signal transduction in plants. Multiple reports have shown that PYLs can improve plant sensitivity to drought, osmotic stress, and cold heat stress [73,74,75,76,77,78]. In our study, the expression of two (CsaV3_3G033450, CsaV3_5G000620, CsaV3_5G010560, and CsaV3_6G001990) and one (CsaV3_4G026530) PYL genes was upregulated in the LC24 vs. L24 and in both the LC3 vs. L3 and LC24 vs. L24 pairwise comparisons, respectively (Tables S2–S5). Among them, 3 PLY genes (CSAV3_4G026530, CSAV3_5G010560, and CSAV3_6G001990) were clustered in the most represented profile 39 (*p* < 0.05) across LC0, LC3, LC12, and LC24 (Figure 7B). We concluded that exogenous COS could alleviate the damage caused by cold stress by activating PYL expression.

## 4. Materials and Methods

### 4.1. Plant Material

In this study, a cold-sensitive cultivar, cucumber “Jinyan No. 4” from Shenyang Agriculture University, was used as the test material. Cucumber seeds were immersed at 55 °C for 10–15 min in water (hot water treatment, HWT) and placed in a moist petri dish for germination in the dark at 28 °C. The sprouting seeds were transferred into a seedling tray in an incubator at a temperature of 28 °C/18 °C (day/night) and a photoperiod of 12 h/12 h (day/night). At the first-true-leaf stage, the robust seedlings were placed in a 7 cm diameter nutrient bowl. The exogenous substance application treatments started when the seedlings developed their third true leaf; each treatment had 20 seedlings, and the biology was repeated three times. Tween 20 (0.02% *v/v*) (Sigma-Aldrich, St. Louis, MO, USA)was used as a surfactant at application time. This experiment was conducted from June 2020 to July 2021 in the Laboratory of Vegetable Cultivation and Ecology Research Group at the Shenyang Agricultural University. 

### 4.2. Sample Treatments

Seedlings at the third-true-leaf stage were pretreated with different concentrations of exogenous GB, CTS, and COS (Sigma-Aldrich, St. Louis, MO, USA), or distilled water at normal temperature (28 °C/18 °C, day/night) for 24 h, after which they were exposed to cold stress (12 °C/6 °C, day/night). The cucumber seedlings were exposed to the following treatments: NK (distilled water-sprayed group, normal temperature), LK (distilled water-sprayed group, cold stress), GB5 (5 mM GB-sprayed group, cold stress), GB10 (10 mM GB-sprayed group, cold stress), GB25 (25 mM GB-sprayed group, cold stress), CTS25 (25 mg·L^−1^ CTS-sprayed group, cold stress), CTS50 (50 mg·L^−1^ CTS-sprayed group, cold stress), CTS100 (100 mg·L^−1^ CTS-sprayed group, cold stress), COS25 (25 mg·L^−1^ COS-sprayed group, cold stress), COS50 (50 mg·L^−1^ COS-sprayed group, cold stress), COS100 (100 mg·L^−1^ COS-sprayed group, cold stress). The fully expanded second and third leaves were sampled for physiological and biochemical analyses of cucumber seedlings.

In the second experiment, based on the results obtained for the first experiment above, seedlings at the third-true-leaf stage were pretreated with the optimal concentrations of exogenous GB, CTS, and COS, or distilled water, at normal temperature (28 °C/18 °C, day/night) for 24 h, and then exposed to cold stress (12 °C/6 °C, day/night). The cucumber seedlings were exposed to the following treatments: NK, LK, NB (10 mM GB-sprayed group, normal temperature), LB (10 mM GB-sprayed group, cold stress), NT (50 mg·L^−1^ CTS-sprayed group, normal temperature), LT (50 mg·L^−1^ CTS-sprayed group, cold stress), NO (50 mg·L^−1^ COS-sprayed group, normal temperature), LO (50 mg·L^−1^ COS-sprayed group, cold stress). The fully expanded second and third leaves were sampled for physiological and biochemical analyses of cucumber seedlings. 

Third-true-leaf seedlings were pretreated with the exogenous substance that was found to be the most effective in the second experiment, 50 mg·L^−1^ COS, or distilled water, at normal temperature (28 °C/18 °C, day/night) for 24 h, and then exposed to cold stress (12 °C/6 °C, day/night) for 0, 3, 12, and 24 h. The cucumber seedlings were exposed to the following treatments: LC0 (50 mg·L^−1^ COS-sprayed group, cold stress for 0 h), LC3 (50 mg·L^−1^ COS-sprayed group, cold stress for 3 h), LC12 (50 mg·L^−1^ COS-sprayed group, cold stress for 12 h), LC24 (50 mg·L^−1^ COS-sprayed group, cold stress for 24 h), L0 (distilled water-sprayed group, cold stress for 0 h), L3 (distilled water-sprayed group, cold stress for 3 h), L12 (distilled water-sprayed group, cold stress for 12 h), and L24 (distilled water-sprayed group, cold stress for 24 h). The fully-expanded second and third leaves were sampled for transcriptome analysis of cucumber seedlings. 

### 4.3. Morphological, Physiological, and Biochemical Analyses

The plant height, stem diameter, fresh weight, dry weight, and seedling index were determined according to the method described in [79]. Root trait phenotyping was performed using the WinRHIZO root-scanning method [80]. The relative water content was determined according to the method described in [81]. The Ci, gsw, Pn, E, and Fv/Fm of the second fully expanded leaf were determined using a photosynthesis system LI-6800 (LiCor Inc., Lincoln, NE, USA) between 8 and 11 am. The SPAD value was determined using a SPAD-502 m (Konica Minolta, Tokyo, Japan). The REC was measured using an ORION TDS conductance meter (ORION Research, Inc., Franklin, MA, USA). The MDA, SS, SP, and Pro contents were determined using the barbiturate coloring (TBA method), anthrone-, Coomassie bright-blue-, and acid ninhydrin methods, respectively. The activities of SOD, POD, and CAT were measured by the nitrogen blue tetrazole photochemical reduction method, the guaiacol oxidation colorimetric method, and the colorimetry method, respectively. 

### 4.4. RNA Isolation, Library Construction, and Illumina Sequencing

Approximately 0.1 g of cucumber seedling leaves were ground to powder with liquid nitrogen. Total RNA was isolated and purified using TRIzol reagent (Invitrogen, Carlsbad, CA, USA), following the manufacturer’s instructions. The RNA amount and purity of each sample were quantified using NanoDrop ND-1000 (NanoDrop, Wilmington, DE, USA). The RNA integrity was detected using a Bioanalyzer 2100 (Agilent, CA, USA) with a RIN number >7.0 and confirmed by electrophoresis on a denaturing agarose gel. After total RNA was extracted, mRNA was purified from total RNA (5 µg) using Dynabeads Oligo (dT) (Thermo Fisher Scientific, CA, USA) with two rounds of purification. Following purification, the mRNA was fragmented into short fragments using divalent cations under elevated temperatures with a Magnesium RNA Fragmentation Module (NEB, cat.e6150, Beverly, MA, USA) at 94 °C for 5–7 min. The cleaved RNA fragments were reverse-transcribed to generate cDNA using SuperScript^TM^ II Reverse Transcriptase (cat. 1896649, USA). The average insert size of the final cDNA library was 300 ± 50 base pairs (bp). Finally, we performed 2 × 150 bp paired-end sequencing (PE150) on an Illumina Novaseq^TM^ 6000 (LC-Bio Technology Co., Ltd., Hangzhou, China), following the manufacturer’s recommended protocol.

### 4.5. Bioinformatics Analysis of RNA-Sequencing (RNASseq)

Cutadapt (https://cutadapt.readthedocs.io/en/stable/(accessed on 20 April 2021), version: cutadapt-1.9) was used to filter the reads that contained adaptor contamination, low-quality bases, and undetermined bases with default parameters. HISAT2 (https://ccb. jhu. edu/software/hisat2/(accessed on 22 April 2021), version: hisat2-2.2.1) was used to map the reads to the cucumber (Chinese Long) genome V3 database (http://cucurbitgenomics.org/organism/20 (accessed on 22 April 2021)). The mapped reads of each sample were assembled using StringTie (https://ccb.jhu.edu/software/stringtie/(accessed on 27 April 2021), version: stringtie-2.1.6) with the default parameters. Transcriptomes from all samples were merged to reconstruct a comprehensive transcriptome using gffcompare (http://ccb.jhu.edu/software/stringtie/gffcompare.shtml (accessed on 30 April 2021), version: gffcompare-0.9.8). After the final transcriptome was generated, StringTie and ballgown (http://www.bioconductor.org/packages/release/bioc/html/ballgown.html (accessed on 5 May 2021)) were used to estimate the expression levels of all transcripts and perform expression abundance for mRNAs by calculating the FPKM. Differential gene expression analysis was performed using DESeq2 software between two different groups (and by edgeR between two samples). Genes with a false discovery rate (FDR) below 0.05 and an absolute fold change ≥2 were considered differentially expressed genes. Differentially expressed genes were then subjected to enrichment analyses of the GO [82] and KEGG [83] pathways. 

### 4.6. Quantitative Real-Time PCR (qRT-PCR)

The total RNA used for RNA-Seq was used for first-strand cDNA synthesis according to the protocol supplied by the manufacturer of the FastKing RT Kit (with gDNase) (Tiangen Biotech, Beijing, China). Quantitative Real-Time PCR and melting curve analyses were performed following the manufacturer’s instructions for SuperReal PreMix Plus (SYBR Green) (Tiangen Biotech, Beijing, China) using a QuantStudio 6 PCR system (Thermo Fisher Scientific, Waltham, MA, USA) with three independent biological replicates. Gene-specific primers were designed using Primer Premier 6 (Table S15). Relative expression levels were calculated using the 2^−∆∆Ct^ method, with *Ubquitin* as the internal control [84].

### 4.7. Statistical Analysis

Data were statistically analyzed using analysis of variance (ANOVA), and treatments were compared using Tukey’s test (*p* < 0.05). 

## 5. Conclusions

This study suggests that low-temperature stress significantly affects the morphology, growth, and physiological and biochemical indices of cucumber seedlings. Different concentrations of exogenous GB, CTS, and COS reduced the cold damage state of cucumber seedlings to varying degrees. The alleviation effect of exogenous GB, CTC, and COS on cold stress was concentration-dependent, and 50 mg·L^−1^ COS showed the best activity. In transcriptome data, functional enrichment of the DEGs revealed that some genes involved in “phenylpropanoid biosynthesis”, “MAPK signaling pathway—plant”, “phenylalanine metabolism”, and “plant hormone signal transduction” responded to cold stress in cucumber seedlings pretreated with exogenous 50 mg·L^−1^ COS at low temperature. Our study is the first to comprehensively analyze the mechanism by which exogenous COS enhances cucumber cold tolerance at physiological and transcriptional levels.

## Figures and Tables

**Table 1:** Effects of exogenous substances on cucumber seedling growth under low-temperature stress.

Treatments	Plant Height (cm)	Stem Diameter (mm)	Ground Fresh Weight (g)	Ground Dry Weight (g)	Underground Fresh Weight (g)	Underground Dry Weight (g)
NK	16.57 ± 0.29 a	4.27 ± 0.09 a	7.90 ± 0.36 a	0.68 ± 0.01 a	1.45 ± 0.07 a	0.10 ± 0.058 a
LK	13.30 ± 0.38 e	3.24 ± 0.05 e	5.31 ± 0.15 bcd	0.53 ± 0.01 bc	1.03 ± 0.11 c	0.07 ± 0.001 bc
GB5	14.57 ± 0.52 cd	3.83 ± 0.06 bcd	5.23 ± 0.09 cde	0.53 ± 0.06 bc	1.25 ± 0.03 abc	0.07 ± 0.003 bc
GB10	16.00 ± 0.52 ab	3.94 ± 0.02 bc	5.99 ± 0.13 bc	0.61 ± 0.04 abc	1.30 ± 0.14 abc	0.09 ± 0.06 ab
GB25	14.37 ± 0.37 cd	3.66 ± 0.03 cd	5.41 ± 0.16 bcd	0.56 ± 0.04 bc	1.25 ± 0.04 abc	0.07 ± 003 bc
CTS25	13.87 ± 0.43 cde	3.82 ± 0.07 bc	5.11 ± 0.02 de	0.51 ± 0.06 c	1.17 ± 0.03 bc	0.06 ± 0.005 c
CTS50	13.97 ± 0.20 cde	3.95 ± 0.13 bc	5.43 ± 0.28 bcd	0.55 ± 0.03 bc	1.30 ± 0.06 abc	0.09 ± 0.01 ab
CTS100	13.97 ± 0.58 cde	3.67 ± 0.10 cd	4.50 ± 0.32 e	0.53 ± 0.02 bc	1.20 ± 0.09 bc	0.08 ± 0.05 abc
COS25	14.23 ± 0.62 cde	3.59 ± 0.21 d	5.63 ± 0.09 bcd	0.57 ± 0.02 bc	1.17 ± 0.07 abc	0.07 ± 0.003 bc
COS50	15.07 ± 0.47 bc	3.99 ± 0.09 ab	6.06 ± 0.18 b	0.62 ± 0.03 ab	1.40 ± 0.07 ab	0.09 ± 0.006 ab
COS100	13.53 ± 0.23 de	3.94 ± 0.04 bc	5.87 ± 0.43 bcd	0.60 ± 0.03 abc	1.11 ± 0.01 c	0.08 ± 0.008 ab

Means in the same category followed by different letters indicate significant differences at *p* < 0.05 using Tukey’s test. The data represent the means of replications ± SD.

**Table 2:** Summary of RNA-Seq data.

Sample	Raw Data	Valid Data	Valid Ratio	Q20%	Q30%	GC Content%	Mapped Reads	Unique Mapped Reads
LC0_1	42,126,804	41,188,626	97.77	99.99	98.72	43.50	40,359,532	34,784,941
LC0_2	43,493,534	42,687,216	98.15	99.99	98.73	44.00	41,817,716	35,960,624
LC0_3	50,654,312	49,472,472	97.67	99.99	98.70	44.00	48,569,549	41,786,964
LC3_1	40,601,628	39,681,714	97.73	99.99	98.64	42.50	38,854,745	33,562,509
LC3_2	39,968,552	39,014,400	97.61	99.99	98.58	43.00	38,175,803	33,057,422
LC3_3	45,104,026	43,962,284	97.47	99.99	98.58	42.50	42,832,193	36,969,503
LC12_1	40,735,332	39,647,658	97.33	99.97	97.83	43.50	38,561,473	32,787,604
LC12_2	46,820,294	45,474,216	97.13	99.99	98.70	44.00	44,594,127	38,804,478
LC12_3	40,971,728	40,014,628	97.66	99.98	98.58	43.50	39,128,642	33,963,021
LC24_1	50,827,142	49,679,218	97.74	99.97	97.83	43.00	48,135,263	40,328,014
LC24_2	49,186,364	47,684,894	96.95	99.97	97.81	42.50	45,853,350	38,741,200
LC24_3	46,031,094	44,790,962	97.31	99.97	97.90	42.50	43,332,675	36,565,195
L0_1	41,077,610	40,257,174	98.00	99.99	98.55	43.00	39,308,565	33,687,253
L0_2	43,754,034	42,721,610	97.64	99.99	98.63	43.00	41,684,077	35,793,435
L0_3	44,196,938	43,303,516	97.98	99.99	98.58	43.50	42,289,716	36,321,136
L3_1	39,312,430	38,223,758	97.23	99.99	98.73	44.00	37,459,192	32,346,810
L3_2	38,853,612	37,929,152	97.62	99.99	98.70	44.00	37,171,141	32,123,659
L3_3	41,441,694	40,526,978	97.79	99.99	98.60	44.00	39,660,858	34,218,611
L12_1	39,318,436	38,390,548	97.64	99.99	98.70	44.00	37,604,628	32,743,831
L12_2	36,926,050	36,050,098	97.63	99.98	97.99	44.50	35,133,412	29,903,919
L12_3	39,152,442	38,130,792	97.39	99.98	97.94	44.50	37,170,463	31,603,798
L24_1	37,801,080	36,925,402	97.68	99.97	98.60	44.00	36,196,280	31,135,293
L24_2	49,462,012	48,542,266	98.14	99.98	98.69	44.00	47,587,324	41,034,627
L24_3	52,902,880	51,657,496	97.65	99.97	97.90	43.50	50,157,853	42,120,150

## Data Availability

The datasets presented in this study can be found in online repositories. The names of the repository/repositories and accession number(s) can be found below: GEO accession numbers GSE210703 and GSE224757.

## Supplementary Materials

The following supporting information can be downloaded at: https://www.mdpi.com/article/10.3390/ijms24076202/s1.

## Abbreviations

GB	Glycine betaine
CTS	Chitosan
COS	Chitosan oligosaccharide
ABA	Abscisic acid
JA	Jasmonic acid
MT	Melatonin
SA	Salicylic acid
PA	Polyamine
Ci	Intercellular CO_2_
gsw	Stomatal conductance
Pn	Net photosynthesis
E	Transpiration rate
REC	Relative electrical conductivity
MDA	Malondialdehyde
SP	Soluble protein
SS	Soluble sugar
Pro	Proline
SOD	Superoxide dismutase
CAT	Catalase
POD	Peroxidase
RNA-Seq	RNA sequencing
DEGs	Differentially expressed genes
