# Supplementary material for: Integrated Physiological and Transcriptomic Analyses Revealed Improved Cold Tolerance in Cucumber (Cucumis sativus L.) by Exogenous Chitosan Oligosaccharide
Source: Int J Mol Sci. 2023 Mar 25;24(7):6202. doi: 10.3390/ijms24076202 (PMC10094205; doi:10.3390/ijms24076202)
Supplement: Supplementary file 1 [file ijms-24-06202-s001.zip › Additional file 1.pdf]

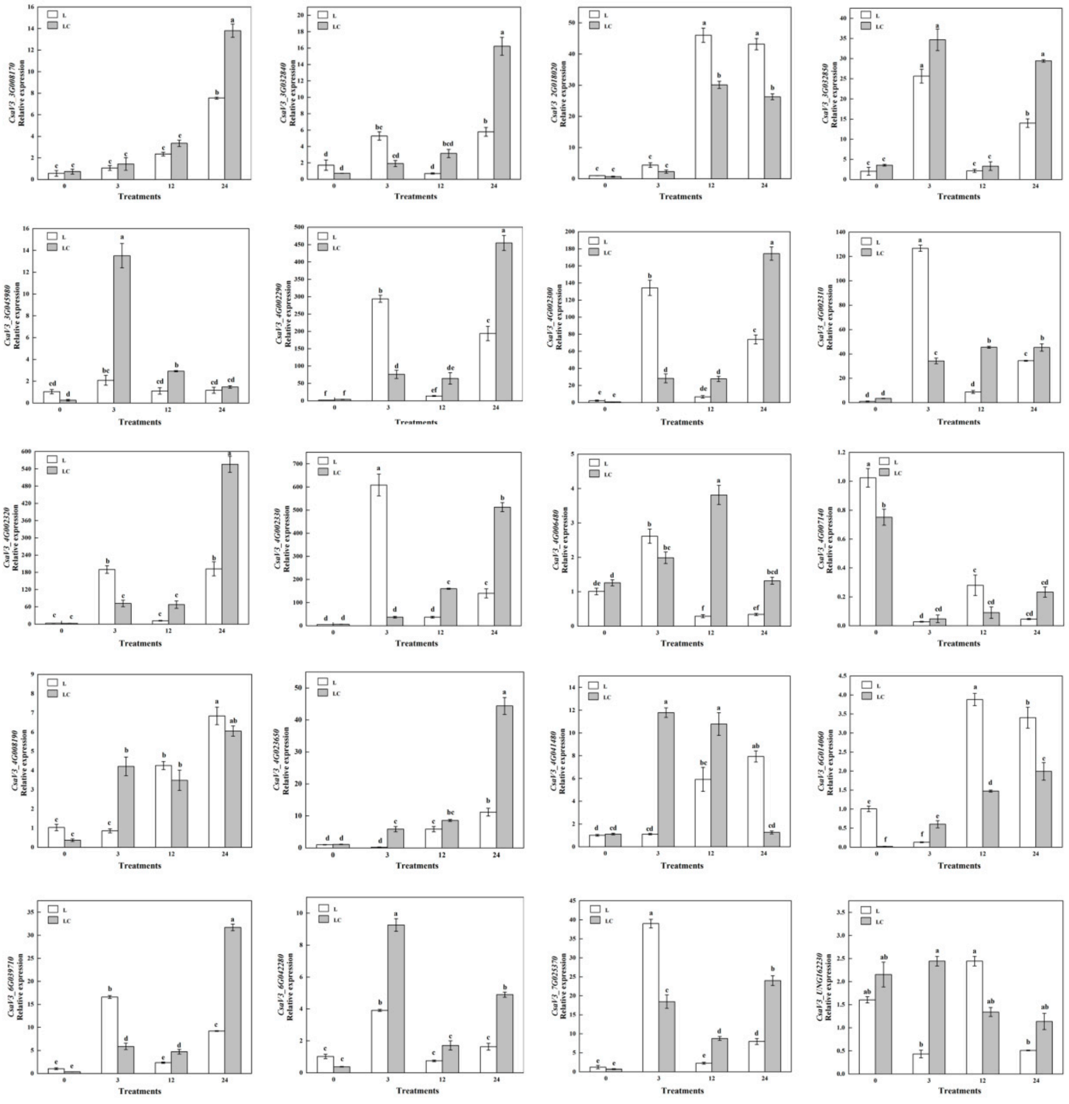

Figure S1. qRT–PCR validation of the DEGs of RNA-seq data.

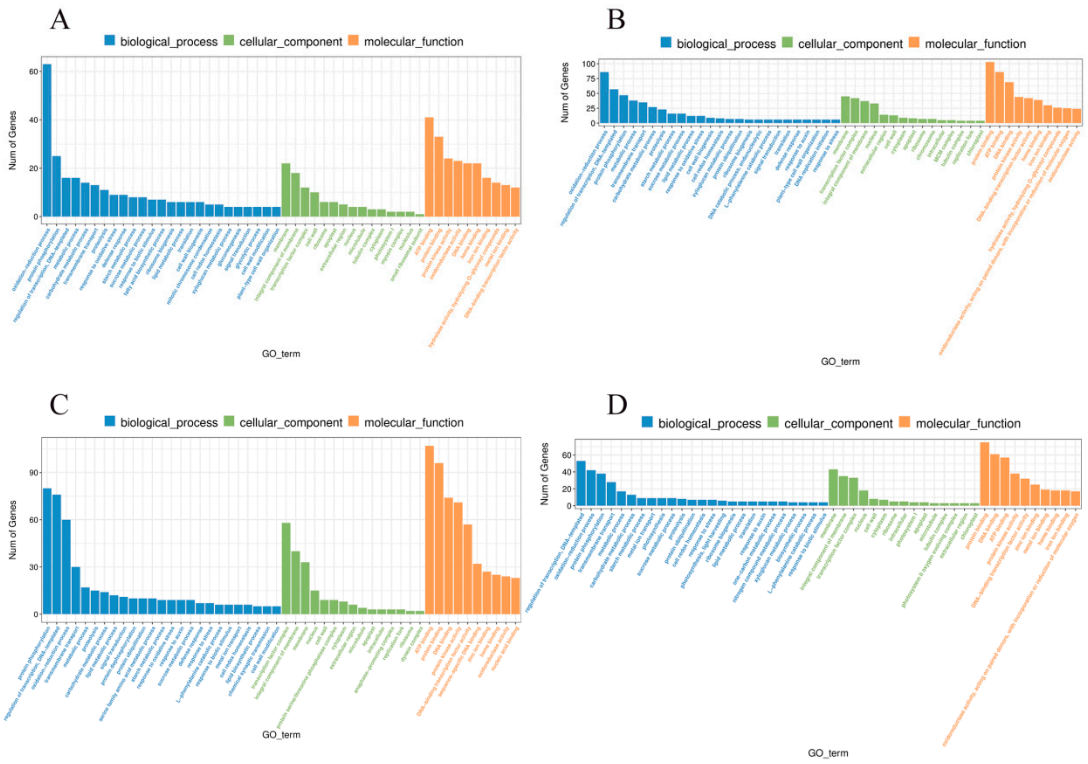

Figure S2. GO analysis of DEGs in (**A**) LC0 vs. L0, (**B**) LC3 vs. L3, (**C**) LC12 vs. L12, (**D**) LC24 vs. L24 pairwise comparison. The Y and X axes correspond to GO terms and the number of DEGs.

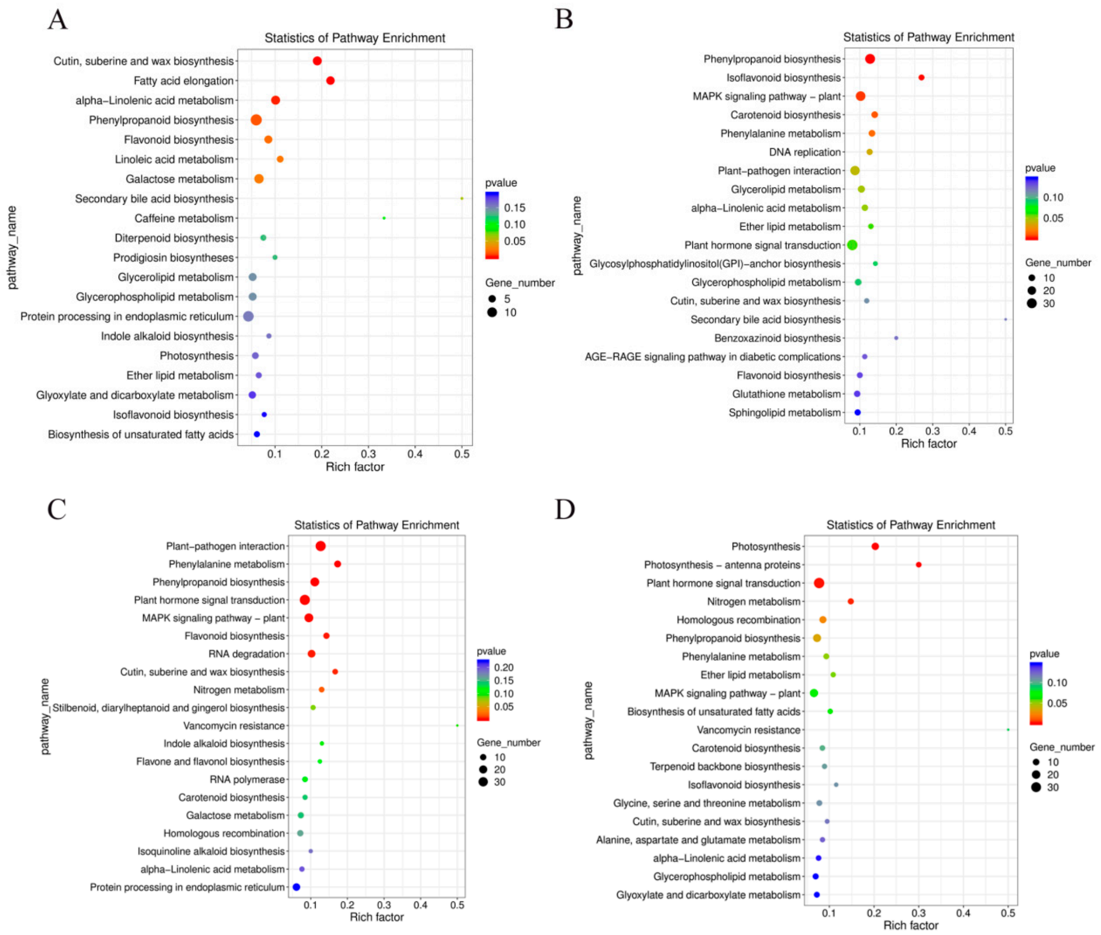

Figure S3. KEGG enrichment analysis of DEGs in (**A**) LC0 vs. L0, (**B**) LC3 vs. L3, (**C**) LC12 vs. L12, (**D**) LC24 vs. L24 pairwise comparison. The Y-axis corresponds to the KEGG pathway, and the X-axis shows the enrichment ratio between the number of DEGs enriched in a particular pathway. The color of the dot represents the q-value, and the size of the dot represents the number of DEGs mapped to the referent pathway.

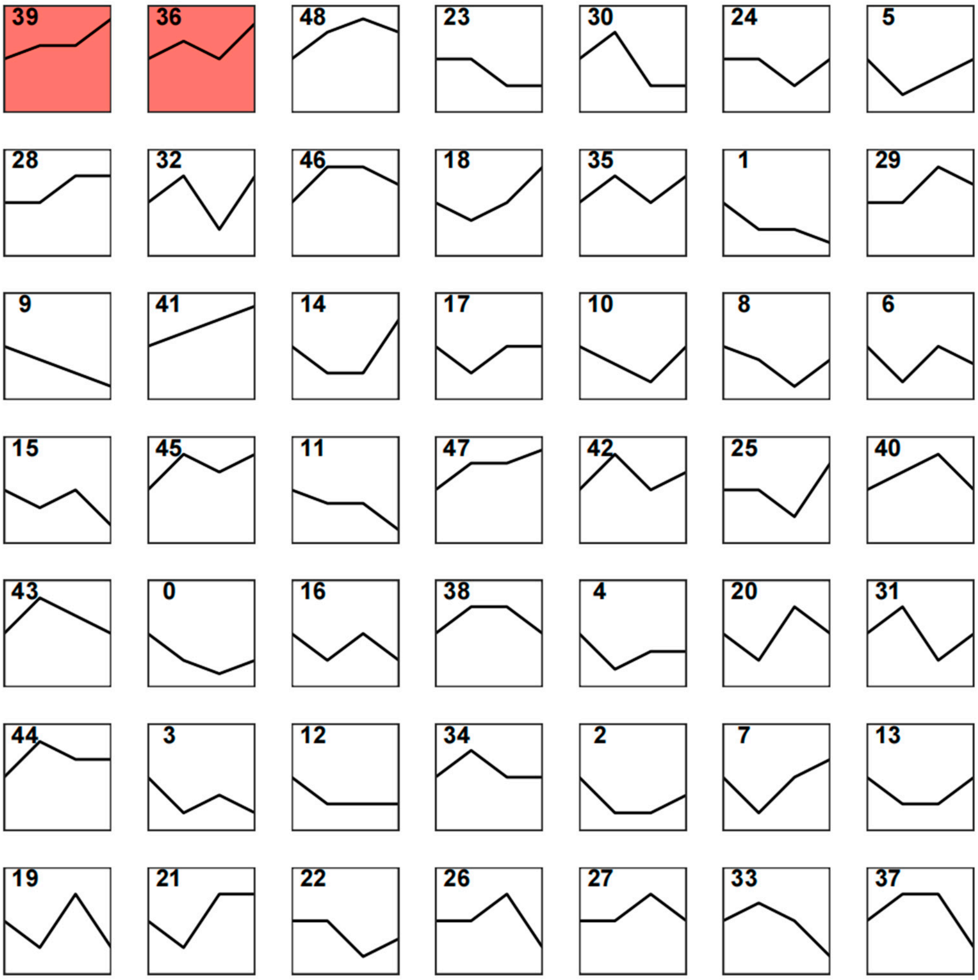

Figure S4. The expression tendency of the DEGs across four treatments (LC0, LC3, LC12, and LC24) inferred by STEM analysis.
